# Single-Step Extraction of *Chlorella vulgaris* Protein by Coupling Cell Disruption and Alkaline-Assisted Solubilization for Enhanced Recovery

**DOI:** 10.3390/foods15213768

**Published:** 2026-10-22

**Authors:** Jun Wei Ng, Tong Mei Teh, Chee Fan Tan, Xuezhi Bi, Felicia Ong, Md. Mahabubur Rahman Talukder

**Affiliations:** 1 Singapore Institute of Food and Biotechnology Innovation (SIFBI), Agency for Science, Technology and Research (A*STAR), 31 Biopolis Way, Nanos, Singapore 138669, Singapore; ng_jun_wei@a-star.edu.sg (J.W.N.); teh_tong_mei@a-star.edu.sg (T.M.T.); 2 Bioprocessing Technology Institute (BTI), Agency for Science, Technology and Research (A*STAR), 20 Biopolis Way, Centros, Singapore 138668, Singapore; tan_chee_fan@a-star.edu.sg (C.F.T.); bi_xuezhi@a-star.edu.sg (X.B.); felicia_ong@a-star.edu.sg (F.O.)

**Keywords:** microalgal protein, single-step extraction, bead milling, high-pressure homogenization, alkaline solubilization, functional properties

## Abstract

*Chlorella vulgaris* (*CV*) is a promising sustainable protein source; however, its rigid cell wall limits protein extraction efficiency. To address this challenge, we previously developed a two-step process involving dry bead milling (BM) followed by alkaline extraction, which achieved a protein solubilization yield of 47.3% using 0.1 M sodium hydroxide (NaOH). To further improve protein recovery, reduce alkaline consumption, and simplify processing, the present study developed single-step extraction strategies that integrate cell disruption and protein solubilization in a single operation. Among the evaluated approaches, a single-step high-pressure homogenization (HPH) process coupled with 0.1 M NaOH achieved a protein solubilization yield of 80.2% after two passes (~5 min, 35,000 psi), representing a 1.4-fold and 1.7-fold increase compared with the single-step BM process (wet BM, 1 h) and the previously reported two-step process (2 h), respectively. Protein functional properties were influenced by the extraction method, with the single-step BM extract showing superior water absorption capacity, foaming capacity, and emulsifying performance. Collectively, the single-step HPH process maximized protein extraction yield and process efficiency, whereas the single-step BM process enhanced protein functionalities. These findings advance the efficient utilization of *CV* as a sustainable alternative protein source.

## 1. Introduction

The global demand for protein has increased substantially over recent decades, driven by rapid population growth, urbanization, and dietary shifts toward protein-rich diets. With the global population projected to reach nearly 10 billion by 2050 [1], meeting nutritional needs in a sustainable manner has become increasingly critical. Conventional protein sources, such as meat and dairy, place considerable pressure on land, water, and energy resources, contributing to greenhouse gas emissions, deforestation, and soil degradation, hence underscoring the need for sustainable alternatives.

Microalgae, particularly *Chlorella vulgaris* (*CV*), have emerged as promising and sustainable sources of edible protein due to their rapid growth rates, high productivity, minimal land requirements, and reduced freshwater demand. In addition, *CV* contains a rich amino acid profile and a high protein level ranging from 48 to 60% [2,3,4]. Despite these advantages, efficient protein extraction remains challenging due to the presence of a rigid cell wall and the limited solubility of intracellular proteins in water. Mild alkaline water (up to pH 9.5), industrially used for plant cell permeabilization and protein solubilization [5], is generally ineffective for microalgae biomass as it does not sufficiently disrupt the robust cell wall structure, thereby hindering protein accessibility and extraction efficiency.

To overcome these limitations, effective cell disruption must be coupled with protein solubilization to facilitate protein release from microalgal cells. Protein extraction from microalgae typically involves two sequential operations: (i) cell disruption to break the rigid cell wall and release intracellular contents, followed by (ii) protein solubilization or extraction of proteins using alkaline or other extraction media. Previous studies by our group investigated sequential protein extraction strategies involving separate cell disruption and alkaline-assisted extraction steps, as well as the recovery and functional characterization of *CV* protein extracts [6,7]. A two-step extraction method comprising bead milling (BM) followed by alkaline solubilization achieved a significantly higher protein solubilization yield (47.3%) than alkaline solubilization alone (16.8%) [6]. Furthermore, the resulting protein extracts exhibited favorable functional properties compared to commercial soy protein isolate [7]. However, despite these advantages, the two-step process remains time-consuming and economically less attractive due to its operational complexity, increased equipment utilization, and the higher risk of product loss arising from multiple processing steps.

Process intensification through the integration of multiple unit operations into a single process step has therefore attracted increasing interest as a strategy to improve bioprocess efficiency and industrial implementation. In this context, the present work extends our previous studies by evaluating a process-intensified extraction strategy in which cell disruption and protein solubilization are integrated into a single operation. Specifically, BM and HPH were assessed within this single-step framework and compared with the previously developed two-step process as a benchmark. Unlike the conventional sequential approach, the integrated strategy eliminates the need for an intermediate separation stage, potentially simplifying the extraction workflow and reducing processing time and chemical inputs. Therefore, the objective of this study was not merely to evaluate another protein extraction method, but rather to determine whether integrating cell disruption and protein solubilization into a single operation could outperform the current state-of-the-art two-step sequential process in terms of protein recovery and process efficiency while maintaining the functionality of the recovered protein extracts. This comparison is particularly relevant because the two-step process represents the closest technological antecedent, allowing the specific benefits arising from process integration to be isolated and quantitatively evaluated. Accordingly, particular attention was given to evaluating protein recovery, processing time, alkali consumption, and preservation of protein functionality as indicators of the benefits arising from process integration.

## 2. Materials and Methods

### 2.1. Materials

The dried *CV* microalgae biomass was obtained from GO SUPERFOODS LTD (Sheffield, UK) and used without further treatment. The biomass contains approximately 54.0% protein, 16.8% fat, 16.9% carbohydrates, 8.03% ash and 4.25% moisture, as previously characterized by our group. Bradford reagent and bovine serum albumin (BSA) were supplied by Sigma-Aldrich (St. Louis, MO, USA), while all other chemicals used in the study were of analytical grade. Ultrapure water was used for all solution preparations.

### 2.2. Preparation of Biomass Suspension

The *CV* biomass was suspended directly in alkaline solution prepared using NaOH. The biomass concentration was adjusted to 50 g L^−1^ to form a homogeneous slurry by mixing at 500 rpm for 30 min at room temperature using an IKA RCT B S105 hot plate magnetic stirrer (IKA, Staufen im Breisgau, Germany). The suspension was gently mixed to ensure uniform dispersion before processing.

### 2.3. Single-Step Extraction

#### 2.3.1. Bead Milling (BM)

The biomass was suspended directly in different NaOH concentrations (0–0.6 M) (Section 2.2) and subjected to BM (Retsch Mixer Mill MM 400 (Retsch, Haan, Germany)) with glass beads of 0.5 mm diameter (Sigma-Aldrich, St. Louis, MO, USA). The cell suspension from a single experimental preparation was divided into eight 50 mL centrifuge tubes. Each tube was filled to 80% of its capacity with 20 mL (~30 g) of glass beads and 20 mL of microalgal cell suspension in NaOH to maintain a solid (glass beads) to liquid (cell suspension) ratio of 1:1 (*v*/*v*). BM was performed at 26 Hz for 1 h with 5 min cooling intervals applied every 10 min [7]. Temperature was maintained below 35 °C during the process. The resulting microalgae biomass suspension was centrifuged at 26,000× *g* for 10 min at a controlled temperature of 20 °C using Sorvall Lynx 4000 centrifuge (Thermo Fisher Scientific, Waltham, MA, USA). The solubilized protein in the supernatant was measured using the Bradford assay as illustrated in Section 2.6. The protein solubilization yield (%) was calculated using the following formula:

$$\mathrm{Protein}\;\mathrm{solubilization}\;\mathrm{yield}\;(\%)\;=\;\frac{\mathrm{Solubilized}\;\mathrm{protein}\;\mathrm{in}\;\mathrm{supernatant}}{\mathrm{Total}\;\mathrm{protein}\;\mathrm{in}\;\mathrm{biomass}\;}\text{ × }100$$

#### 2.3.2. High-Pressure Homogenization (HPH)

The biomass was suspended directly in 0.1 M NaOH (Section 2.2) and subjected to HPH using a Constant Systems CF2 cell disruptor (Constant Systems, Daventry, UK). The HPH was performed at a nominal pressure of 35,000 psi up to 5 passes. The temperature was maintained below 35 °C throughout the process using the integrated cooling jacket of CF2 cell disruptor. The temperature was measured after each pass to ensure that it remained below the specified limit. The homogenized biomass suspension was collected and centrifuged at 26,000× *g* by using a Sorvall Lynx 4000 centrifuge (Thermo Fisher Scientific, Waltham, MA, USA) for 10 min at 20 °C to facilitate the efficient removal of fine cell debris and insoluble materials from the protein-containing supernatant. The solubilized protein in the supernatant was measured using the Bradford assay as illustrated in Section 2.6. The protein solubilization yield (%) was calculated as described in Section 2.3.1.

### 2.4. Two-Step Extraction Comprising Dry BM Followed by Alkaline Solubilization

The two-step BM dataset used for comparison was previously reported in [7]. For the present study, the original replicate-level observations from that work were available and were included directly solely as benchmark. The historical dataset was considered comparable to the newly generated data because the experiments employed the same *CV* biomass source and batch, identical bead milling and extraction procedures, analytical methods, and experimental conditions. All experiments were conducted in the same laboratory under comparable operating conditions.

### 2.5. Single-Step Alkali Extraction Alone

The dried *CV* biomass was suspended in 0.1 M NaOH at a biomass loading of 50 g L^−1^ and stirred at 400 rpm and 37 °C for 1 h. The separation of the supernatant from the pellet was conducted by centrifugation at 26,000× *g* (Sorvall Lynx 4000 centrifuge (Thermo Fisher Scientific, Waltham, MA, USA) for 10 min at 20 °C. The solubilized protein concentration in the supernatant was determined using the Bradford assay as described in Section 2.6. The protein solubilization yield (%) was calculated according to the formula provided in Section 2.3.1.

### 2.6. Protein Determination in Liquid Samples by Bradford Assay

The concentration of solubilized protein in extraction liquors was determined using the Bradford assay [8], which has been widely applied for the quantification of soluble proteins from microalgae extracts [9]. The Bradford method was chosen because it is a sensitive, rapid, and widely used method for determining soluble protein concentrations in aqueous solutions. Although the Dumas method can be applied to liquid samples, it generally requires larger sample quantities and is less suitable for measuring the low concentrations of soluble proteins present in extraction liquors.

Following protein extraction, samples were centrifuged at 26,000× *g* (Sorvall Lynx 4000 centrifuge (Thermo Fisher Scientific, Waltham, MA, USA)) for 10 min, and the supernatant was collected for analysis. Because the extracts were generated under alkaline conditions, samples were diluted 20- to 100-fold with deionized water before analysis to ensure that protein concentrations fell within the calibration range and to minimize the concentration of residual alkali. Based on the applied dilution factors, the estimated residual NaOH concentration in the diluted samples ranged from approximately 1.5 to 7.0 mM for extracts prepared using 0.03–0.7 M NaOH. The pH of the diluted samples was not measured.

Quantification was performed using a BSA calibration curve covering 0.0–1.0 mg mL^−1^, yielding a coefficient of determination (R^2^ = 0.9997). For each determination, 50 µL of diluted sample or standard was mixed with 1.5 mL of Bradford reagent in a 2 mL microcentrifuge tube and incubated for 10 min at room temperature. Subsequently, 250 µL of the reaction mixture was transferred to a 96-well microplate, and absorbance was measured at 595 nm using an Agilent BioTek Synergy HTX microplate reader (Agilent Technologies, Santa Clara, CA, USA). A reagent blank consisting of Bradford reagent and deionized water was included and used for background correction. Technical measurements were performed in triplicate for each independent experimental replicate.

Although matrix-matched standards and formal interference studies were not conducted, the substantial sample dilution, together with the approximately 30-fold excess of acidic Bradford reagent (1.5 mL) relative to sample volume (50 µL), was expected to minimize the influence of residual alkali and potential matrix effects on the assay response.

### 2.7. Sodium Dodecyl Sulfate-Polyacrylamide Gel Electrophoresis (SDS-PAGE) Analysis

The electrophoretic profile of the soluble protein obtained using the single-step BM method was analyzed according to the method described by Laemmli [10] using 12% precast polyacrylamide gel (Bio-Rad, Hercules, CA, USA). Protein samples were mixed with 2× Laemmli sample buffer containing 200 mM dithiothreitol (Bio-Rad, Hercules, CA, USA) and heated at 95 °C for 10 min. Each sample containing 30 μg of protein was loaded onto the gel, and electrophoresis was performed at a constant voltage of 140 V for 45 min using a Mini-PROTEAN Tetra Cell system (Bio-Rad, Hercules, CA, USA). Following electrophoresis, the gel was stained with Coomassie Brilliant Blue R-250 (Sigma-Aldrich, St. Louis, MO, USA) and destained with a methanol/acetic acid/deionized water solution (50:10:40, *v*/*v*/*v*). Protein molecular weights were estimated using a prestained protein ladder covering the range of 10–250 kDa (Thermo Fisher Scientific, Waltham, MA, USA).

### 2.8. Protein Recovery and Drying

The proteins solubilized in NaOH solution were separated by pH-induced precipitation and centrifugation. The pH of the protein solution was adjusted to 4.0 using 1 M hydrochloric acid (HCl) to facilitate protein precipitation. This pH was selected based on our previous optimization study of precipitation pH of *CV* protein [6]. This finding is also consistent with other literature reporting that pH 4.0 is near the isoelectric point of microalgal proteins [11,12], where the net surface charge of the proteins is minimized, resulting in reduced electrostatic repulsion, decreased solubility, and enhanced protein aggregation and precipitation. The precipitated proteins were subsequently collected by centrifugation at 2800× *g* for 10 min at 20 °C using a Sorvall™ Lynx 4000 centrifuge (Thermo Fisher Scientific, Waltham, MA, USA). A lower centrifugation force was used at this stage as the precipitated proteins had formed aggregates that could be readily recovered under these conditions. The precipitated protein was freeze-dried at −56 °C and 0.021 mbar (Labconco, Kansas City, MO, USA).

The total protein content of the freeze-dried extract was measured using the Dumas combustion method in a DUMATHERM instrument (Gerhardt Analytical Systems, Königswinter, Germany). The Dumas method was selected because it quantifies total nitrogen content and is a robust, reliable, and widely accepted approach for protein determination in solid samples, thereby providing a more accurate assessment of protein recovery. In contrast, the Bradford assay is not directly applicable to dried protein products, as it requires complete protein solubilization prior to analysis and may be influenced by incomplete dissolution of the recovered material. A nitrogen-to-protein conversion factor of 6.25 was used as it is commonly applied to microalgal biomass, including *Chlorella* species [13]. It should be noted that the presence of non-protein nitrogen in microalgae may result in some overestimation of absolute protein content. Therefore, while the reported absolute protein contents, purities, and recovery values may be affected by the selected conversion factor, the relative comparisons among extraction treatments remain valid because the same factor was applied consistently across all samples. The protein recovery yield was estimated based on the formula below. This formula was applied to all the above protein extraction methods

$$\mathrm{Protein}\;\mathrm{recovery}\;(\%)=\frac{\mathrm{Protein}\;\mathrm{in}\;\mathrm{dry}\;\mathrm{extract}}{\mathrm{Total}\;\mathrm{protein}\;\mathrm{in}\;\mathrm{biomass}\;}\text{ × }100$$

### 2.9. Functional Properties

#### 2.9.1. Protein Solubility

Protein solubility was determined based on the method of Ma et al. [14] with minor modifications. The protein extract was suspended in deionized water (pH 6.83) to achieve a final concentration of 100 mg mL^−1^. The suspension was vortexed and subsequently shaken horizontally at 250 rpm for 1 h at 22–24 °C. It was then centrifuged at 19,800× *g* for 10 min using an Eppendorf centrifuge 5424 (Hamburg, Germany). The protein content in the supernatant was quantified using the Bradford assay (Section 2.6). Protein solubility was calculated using the following equation:

$$\mathrm{Protein}\;\mathrm{solubility}\;\left(\%\right)=\frac{\mathrm{Supernatant}\;\mathrm{protein}\;\mathrm{content}}{\mathrm{Total}\;\mathrm{protein}\;\mathrm{content}}\;\times \;100$$

#### 2.9.2. Water Absorption Capacity (WAC)

WAC was measured following the method of the American Association of Cereal Chemists Technical Committee [15]. Briefly, deionized water was gradually added to 0.25 g of sample (W1) with continuous vortex mixing to obtain a homogeneous paste. The mixture was then allowed to stand at room temperature for 80 min, followed by centrifugation at 9400× *g* (Eppendorf centrifuge 5424, Hamburg, Germany) for 1 h. The free water droplet was carefully removed, and the slurry weight (W2) was recorded. WAC was determined by the following equation:

$$\mathrm{WAC}\;\left(\%\right)=\frac{W2\;-\;W1}{W1\;}\;\times \;100$$

#### 2.9.3. Oil Absorption Capacity (OAC)

OAC was determined according to the method described by Du et al. [16]. In brief, 0.5 g of protein extract (W) was mixed with 5 g of soybean oil (W1). The mixture was vortexed for 2 min and then shaken at 200 rpm for 30 min. After centrifugation at 9400× *g* (Eppendorf centrifuge 5424, Hamburg, Germany) for 1 h, the free oil was carefully decanted and weighed (W2). OAC was calculated according to the equation below:

$$\mathrm{OAC}\;\left(\%\right)=\frac{W1\;-\;W2}{W}\;\times \;100$$

#### 2.9.4. Emulsifying Properties

The emulsifying activity index (EAI) and emulsion stability index (ESI) of the protein extract were measured according to the method of Pearce and Kinsella [17] with slight modifications. In brief, 6 mL of protein solution (1 mg mL^−1^) was mixed with 2 mL of soybean oil. The mixture was homogenized for 2 min at 10,000 rpm using an Ultra-Turrax homogenizer (T18, IKA, Staufen im Breisgau, Germany) to form a stable emulsion. Subsequently, 50 µL of the emulsion was pipetted from the bottom of the tube at 0 min and 10 min after homogenization and dispersed into 5 mL of 0.1% (*w*/*v*) SDS solution. The absorbance of the mixture at 0 min and 10 min was measured at 500 nm (UV-1800, Shimadzu Co., Kyoto, Japan). EAI and ESI were determined as follows:

$$\mathrm{EAI}\;\left(m^{2}\;g^{-1}\right)=\frac{2.303\;\times \;A_{0\;}\times \;2\;\times \;d}{L\;\times \;10000\;\times \;\varphi \;\times \;C}$$

$$\mathrm{ESI}\;\left(\min\right)=\frac{A_{0}-∆t}{A_{0}-A_{10}}$$

where A_0_ and A_10_ are the absorbance of solution at 0 and 10 min, d is the dilution factor (d = 100), L is the pathlength of the cuvette (L = 1 cm), φ is the oil volume fraction (φ = 0.25), C is the initial protein concentration (C = 0.001 g/mL), and ∆t is the sampling interval (∆t = 10 min). The factor of 10,000 is included as a unit conversion factor to express the EAI in m^2^ g^−1^.

#### 2.9.5. Foaming Properties

The foaming capacity (FC) and stability (FS) of the proteins were determined using the method reported by Timilsena et al. [18]. Briefly, 10 mL of 0.5% (*w*/*v*) sample was prepared using Milli-Q water and homogenized using Ultra-Turrax T18 homogenizer (IKA, Staufen im Breisgau, Germany) for 1 min at 10,000 rpm. The sample volumes before (V1) and after (V2) homogenization were recorded. The homogenized sample was settled at room temperature for 10 min, and the sample volume (V3) was measured. FC and FS were expressed as follows [19]:

$$\mathrm{FC}\;\left(\%\right)=\frac{V2\;-\;V1}{V1}\;\times \;100$$

$$\mathrm{FS}\;\left(\%\right)=\frac{V3-V1}{V2-V1}\times 100$$

### 2.10. Statistical Analysis

All experiments were conducted using three independent experimental replicates (*n* = 3). For each experimental replicate, analytical measurements were performed in triplicate and treated as technical measurements rather than independent observations. Statistical analyses were conducted using SPSS version 28.0 (IBM, Armonk, NY, USA) based exclusively on values obtained from the three independent experimental replicates. Differences among treatments were evaluated by one-way analysis of variance (ANOVA) followed by Tukey’s multiple comparison test, with statistical significance established at *p* < 0.05. Error bars in all figures represent the standard deviation (SD) of the independent experimental replicates. The two-step BM dataset previously reported in [7] was analyzed using the original replicate-level observations generated in that study, which were available to the authors and incorporated directly into the statistical analyses; published mean ± SD values were not used as individual observations. To ensure comparability among treatments, all datasets were generated using the same *CV* biomass batch, identical bead-milling and protein extraction procedures, and the same analytical methods.

## 3. Results and Discussion

### 3.1. Single-Step Protein Extraction from CV Dry Biomass

#### 3.1.1. Single-Step BM

Our previous study showed that a two-step process consisting of dry BM-mediated cell disruption (26 Hz for 1 h) of *CV* biomass followed by alkaline extraction with 0.1 M NaOH for an additional 1 h achieved a protein solubilization yield of 47.3%, whereas alkaline extraction alone resulted in a yield of only 16.8% [6]. Building on these findings, the present study employed the same BM condition (26 Hz, 1 h) in a single-step approach and evaluated protein solubilization across different NaOH concentrations. The NaOH concentration ranges differed between the two studies due to their respective experimental objectives. However, 0.1 M NaOH was included in both studies to enable direct comparison.

**Figure 1:**
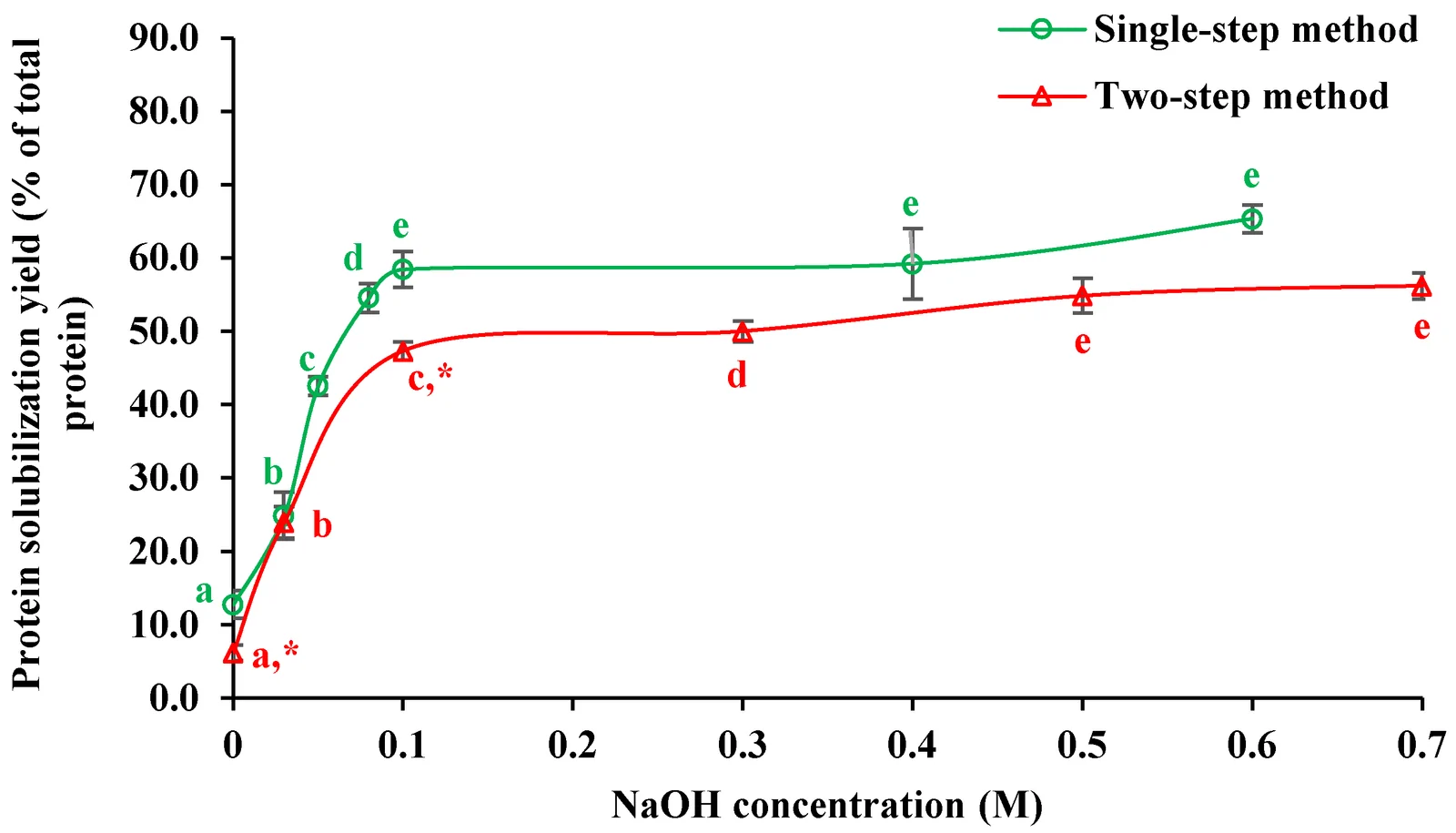
Protein solubilization yield of *CV* obtained using the single-step BM and two-step BM methods at different NaOH concentrations. Two-step BM data were previously reported in [7]. The original replicate data were available to the authors and were incorporated into the statistical analysis. Data are presented as mean ± SD (*n* = 3 independent extraction experiments). Different letters indicate significant differences within the extraction method (*p* < 0.05). Asterisks (*) indicate a significant difference between the single-step and two-step extraction methods at the same NaOH concentration (*p* < 0.05).

**Figure 2:**
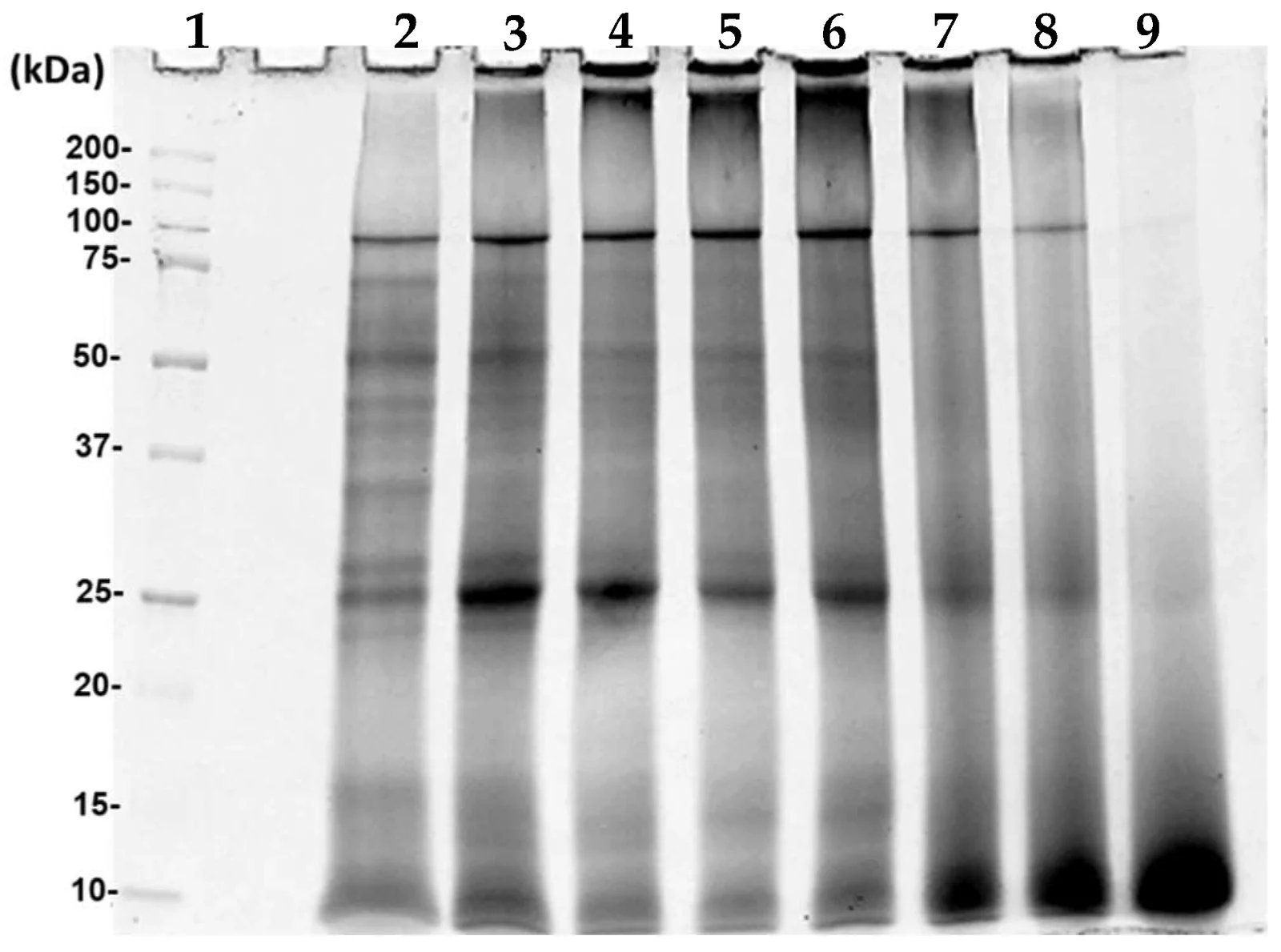
SDS-PAGE profiles of protein extracted from *CV* using the single-step BM method at different NaOH concentrations. Lanes: (1) molecular weight marker (Precision Plus 10–250 kDa, Bio-Rad), with sizes in kDa indicated on the left, (2) 0 M, (3) 0.03 M, (4) 0.05 M, (5) 0.08 M, (6) 0.1 M, (7) 0.4 M, (8) 0.6 M, (9) 1.0 M.

It can be seen from Figure 1 that the single-step method significantly improved the protein solubilization yield at different NaOH concentrations compared to the two-step method [7]. The single-step solubilization yield using 0.1 M NaOH reached 58.5%, which was 1.2-fold higher than the two-step method, highlighting its better efficacy despite reduced processing time (1 h). To achieve a similar yield, two-step process required 7-fold higher NaOH concentration of 0.7 M. Although NaOH concentrations above 0.1 M resulted in further increases in protein solubilization yield for both extraction methods, higher alkali concentrations may induce protein structural changes; however, such changes were not directly evaluated in the present study. As shown in Figure 2, protein profiles obtained at NaOH concentrations below 0.1 M were generally comparable to those observed in the freshwater control (0.0 M NaOH, lane 2). In contrast, increasing the NaOH concentration above 0.1 M resulted in a noticeable decrease in the intensity of several higher-molecular-weight protein bands, accompanied by a more prominent band at approximately 10 kDa. These changes may suggest protein fragmentation or other structural alterations induced by alkaline treatment. However, alternative explanations, including changes in protein solubility, extractability, aggregation behavior, or selective enrichment of specific protein fractions, cannot be excluded based on SDS-PAGE analysis alone.

It should be noted that the primary objective of this study was not to benchmark all available protein extraction technologies but to assess whether process integration could provide advantages over the closest technological antecedent—namely, the dry BM-assisted two-step alkaline extraction process as a benchmark. The two-step BM original replicate data were available to the authors and incorporated into the statistical analysis; published mean ± SD values were not used as individual observations for statistical analysis. Comparing the integrated process with its direct predecessor allows the specific effects of combining cell disruption and extraction into a single operation to be isolated and quantitatively evaluated. Broader comparisons with alternative extraction technologies may be valuable in future studies but were beyond the scope of the present work.

Although the underlying mechanisms were not investigated in the present study, the superior performance of the integrated single-step approach may be associated with processing the biomass in an aqueous environment. The hydrated state of the biomass may facilitate interactions between the extraction medium and cellular components, enhancing protein release and solubilization. Furthermore, the aqueous phase may promote the transfer of released proteins into the surrounding medium, potentially reducing their association with disrupted cell debris and improving their subsequent recovery. However, no direct measurements of cell disruption efficiency, solvent penetration into the cellular matrix, protein distribution among process streams, or other mass-transfer-related phenomena were conducted. Therefore, these explanations should be regarded as mechanistic hypotheses rather than experimentally verified findings. Further studies involving direct characterization of these processes are required to elucidate the mechanisms responsible for the observed improvement in protein recovery.

#### 3.1.2. Single-Step HPH

The single-step method was further extended to HPH under the same alkaline conditions (0.1 M NaOH) and microalgal biomass loading of 50 g L^−1^ to assess its effectiveness over BM. The NaOH concentration of 0.1 M was chosen so that the result can be directly compared with those obtained using BM. The HPH pressure was set at 35,000 psi, which has been previously found to be effective for the disruption of microalgae (*Auxenochlorella protothecoides*) cells in freshwater without NaOH [20]. The protein solubilization yield increased with the number of passes and reached a maximum of 80.2% at the second pass (Table 1). However, further increases in the number of passes resulted in a gradual decline in yield. Similar trends have been reported by Irvani et al. [21] who observed that excessive HPH processing reduced soluble protein recovery of *Nannochloropsis* sp. which was attributed to post-release protein denaturation and aggregation. Other factors such as protein precipitation and the interactions between proteins and other disrupted cellular components may also have contributed to the observed decline. However, further studies are necessary to determine the underlying mechanisms. These findings suggest that two HPH passes were sufficient to maximize *CV* protein recovery whereas additional passes increased processing energy without providing further extraction benefits.

**Figure 3:**
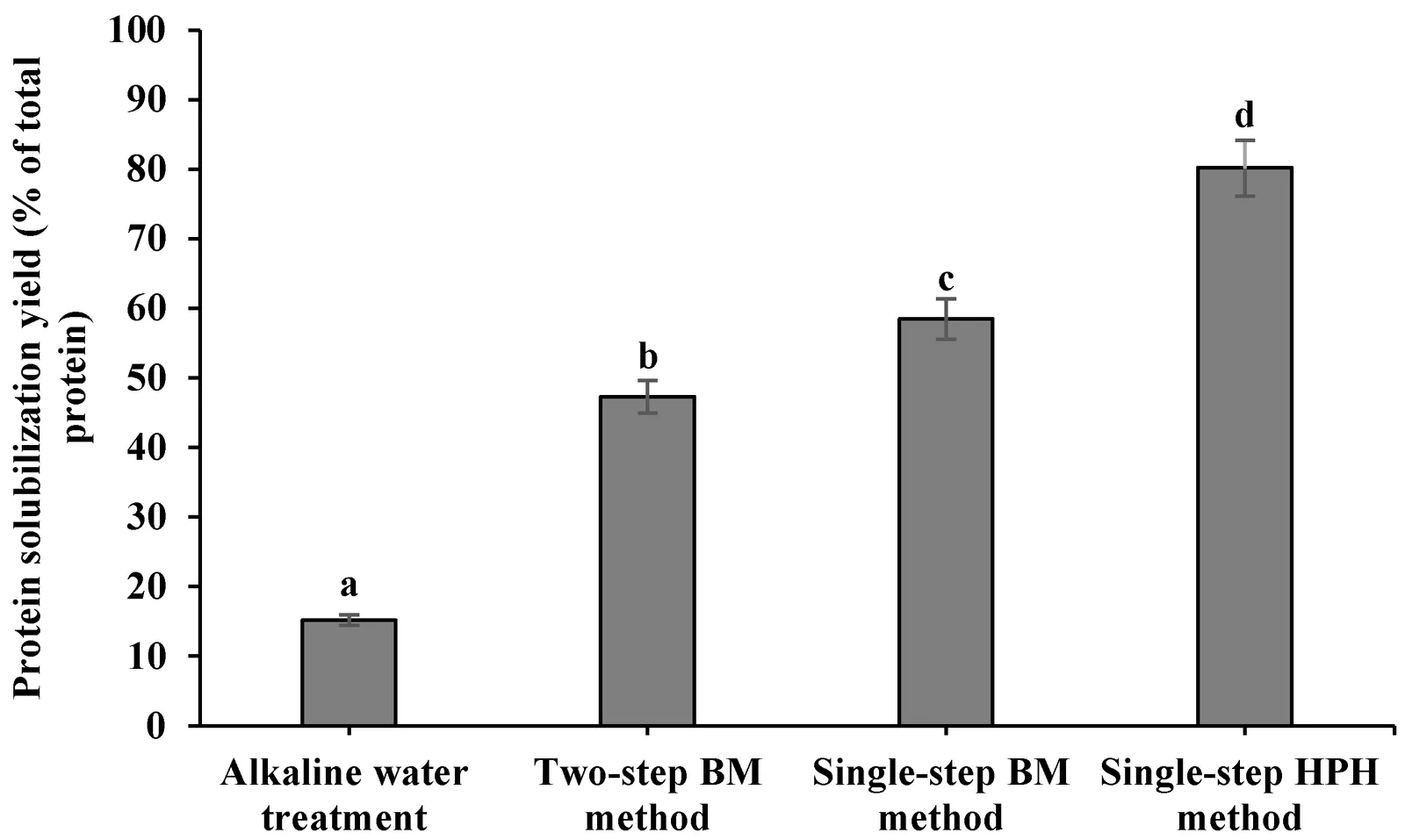
Protein solubilization yield obtained using 0.1 M NaOH across different extraction methods. Two-step BM data were previously reported in [7]. The original replicate data were available to the authors and were incorporated into the statistical analysis. Data are presented as mean ± SD (*n* = 3 independent extraction experiments). Different letters indicate significant differences among the extraction methods (*p* < 0.05).

The single-step HPH protein solubilization yield was compared with different methods as demonstrated in Figure 3. It can be seen that the single-step HPH method outperformed all methods tested. The protein solubilization yield obtained using single-step HPH method (80.2%) was 1.4- and 1.7-fold higher than those of single-step BM (58.5%) and two-step BM (47.3%) method, respectively. It is also obvious that alkaline extraction alone with 0.1 M NaOH was least effective; the solubilization yield reached only 15.2%, which is similar to our previous results (16.8%) [6].

Furthermore, the protein solubilization yield achieved using the proposed single-step HPH method exceeded those reported for *CV* extraction using wet BM in water, including 39.0% reported by Alavijeh et al. [22], 46.0% reported by Liu et al. [23], and 54.0% by Bernaert et al. [24]. Postma et al. [25,26] also reported lower protein solubilization yields of 36.3% and 42.0% when performing wet BM in phosphate-buffered saline. In comparison with HPH-assisted extraction of *CV* protein, the yield obtained in this study was substantially higher than the 51.68% by Safi et al. [27] after two passes and the approximately 58.0% by Carullo et al. [28] after five passes in water. Similarly, Ursu et al. [12] achieved a protein solubilization yield of only 52.0% after two HPH passes at pH 12. Collectively, these results further highlight the superior extraction efficiency and practical advantages of the proposed single-step approach, which combines high protein recovery with relatively mild processing conditions and reduced processing time.

**Figure 4:**
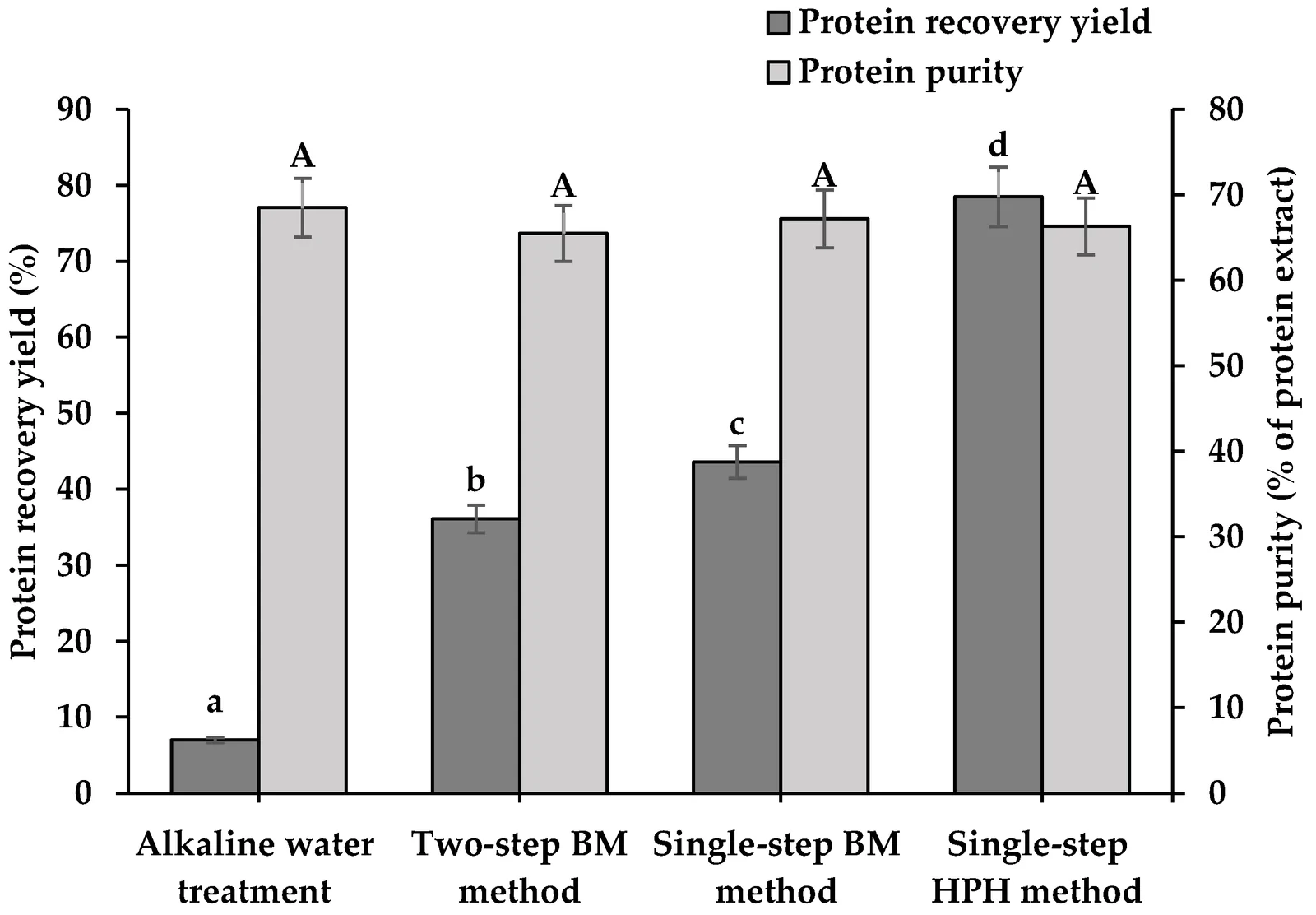
Effects of different methods on protein recovery yield and protein purity of *CV* protein-rich extracts. Two-step BM data were previously reported in [7]. The original replicate data were available to the authors and were incorporated into the statistical analysis. Data are presented as mean ± SD (*n* = 3 independent extraction experiments). Different lowercase letters indicate statistically significant differences in protein recovery yield, whereas different uppercase letters indicate statistically significant differences in protein purity among the extraction methods (*p* < 0.05).

The solubilized protein in alkaline 0.1 M NaOH solution was recovered by pH-induced precipitation and subsequent freeze drying. The protein recovery and purity (protein content in the dry extract) under different methods were demonstrated in Figure 4. Although all methods produced extracts with comparable protein purity (66–68%), substantial differences in protein recovery yield were observed, ranging from 30.0% to 78.5% across the different methods. The single-step HPH method resulted in the highest protein recovery (78.5%), followed by single-step BM (43.6%), two-step BM (36.1%), and alkaline treatment alone (7.0%). These results suggest that the extraction method exerted a greater influence on protein release and recovery than on the final purity of the recovered protein fraction, with HPH-mediated single-step processing being the most effective approach among the methods evaluated under the conditions tested. Previous studies have reported that HPH can promote extensive cell disruption, which may facilitate NaOH penetration into the biomass and enhance protein extraction [29,30]. Furthermore, NaOH promotes the dissociation of protein-cell wall interactions and protein solubilization [31]. Therefore, the combined application of HPH and alkaline treatment in a single-step process may have contributed to the improved protein recovery observed in this study. However, cell disruption was not directly evaluated, and thus these explanations remain speculative. It has been reported that the two-step ultrasonication and chemical (e.g., solvent, H_2_O_2_ and alkali) treatments improved microalgae protein solubilization [32]. Safi et al. [29] compared four different cell disruption techniques and found HPH to be most effective for extracting protein and pigments from microalgae *CV*. In addition to the cell disruption efficiency, variations in protein solubilization, protein denaturation, and downstream processing losses likely contributed to the observed differences in recovery.

The protein recovery values reported in this study represent the proportion of initial biomass protein recovered in the final freeze-dried protein fraction. A complete protein mass balance was not established because protein contents in the insoluble residue, post-precipitation supernatant, and other residual process streams were not quantified. Therefore, the unrecovered protein fraction may be distributed among residual solids, soluble proteins remaining after precipitation, and process-related handling losses. Although the present work demonstrates the relative effectiveness of different extraction strategies, future studies should quantify all process streams to establish a closed protein mass balance and support process scale-up and waste valorization.

The advantages of the proposed single-step alkaline-assisted extraction process extend beyond workflow simplification. The novelty of the present study lies in the systematic evaluation of integrated single-step processing strategies combining cell disruption and alkaline-assisted protein solubilization, including both BM and HPH approaches, and their comparison with conventional processing methods. By integrating cell disruption and protein solubilization into a single operation, intracellular proteins can be released and solubilized simultaneously rather than through sequential processing steps. This integrated strategy eliminates the need for an intermediate separation stage between disruption and extraction, thereby reducing process complexity and potentially minimizing losses associated with intermediate handling and transfer. Compared with the conventional two-step BM approach, which was included as a benchmark for comparison, the single-step processes achieved higher protein recovery and extraction efficiency while requiring fewer processing steps. Overall, the results demonstrate that process integration can improve extraction performance while simplifying the overall workflow. These findings highlight the potential of single-step alkaline-assisted extraction strategies as efficient platforms for the future development and scale-up of microalgal protein extraction processes.

### 3.2. Functional Properties

**Figure 5:**
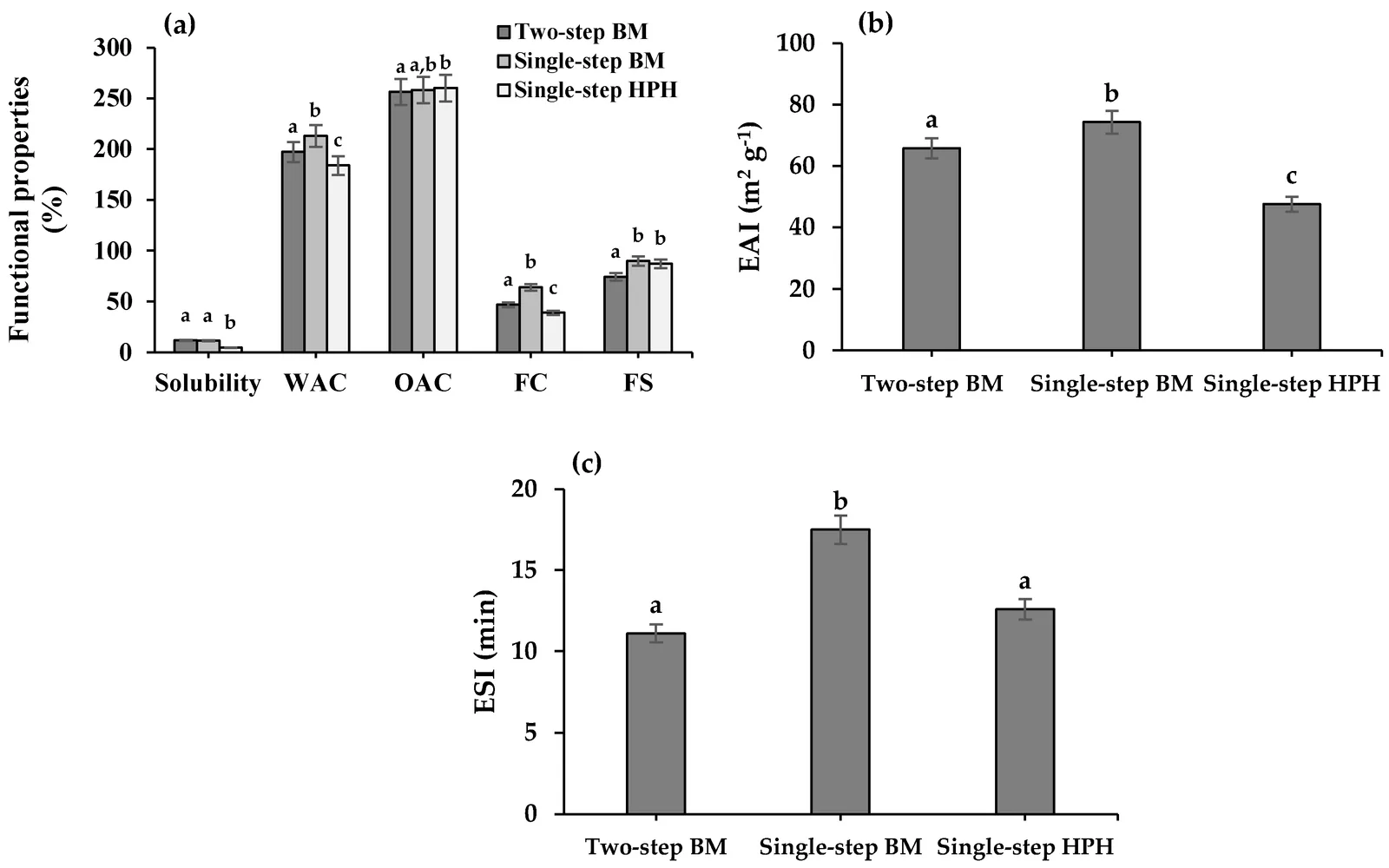
Functional properties of *CV* protein-rich extracts obtained using different methods. (**a**) Solubility, WAC, OAC, FC and FS; (**b**) EAI; and (**c**) ESI. Two-step BM data were previously reported in [7]. The original replicate data were available to the authors and were incorporated into the statistical analysis. Data are presented as mean ± SD (*n* = 3 independent extraction experiments). Different letters within each functional property indicate statistically significant differences among the treatments at *p* < 0.05.

The functional properties of *CV* protein-rich extracts obtained using the single-step HPH process were compared with those produced using single-step BM and two-step BM processes (Figure 5). Data for the two-step BM process were taken from our previous study [7] to facilitate comparison among the protein recovery approaches. Protein-rich extracts recovered through alkaline extraction alone were not included in the analysis because the recovered quantity was insufficient for functional property characterization.

Figure 5 shows the functional properties of protein-rich extracts obtained using the three extraction methods. Protein solubility was generally low for all extracts; however, the single-step HPH extract exhibited significantly lower solubility than the BM-derived extracts (*p* < 0.05) (Figure 5a). WAC differed significantly among all treatments, with the single-step BM extract showing the highest value and the single-step HPH extract showing the lowest value (*p* < 0.05). In contrast, the single-step HPH extract exhibited the highest OAC, which was significantly higher than that of two-step BM extract (*p* < 0.05), while single-step BM extract was comparable to both treatments. FC also varied significantly among the extraction methods, with single-step BM producing the highest FC and single-step HPH the lowest (*p* < 0.05). In contrast, FS was not significantly affected by the extraction method (*p* > 0.05). These results indicate that the extraction strategy influenced several functional attributes of the protein-rich extracts, although the magnitude of the effect varied among the evaluated properties.

More pronounced differences were observed in the emulsifying properties of the protein-rich extracts (Figure 5b,c). Single-step BM exhibited the highest EAI (74.3 m^2^ g^−1^), followed by two-step BM (65.8 m^2^ g^−1^), whereas single-step HPH showed the lowest EAI (47.6 m^2^ g^−1^), with all treatments differing significantly from one another (*p* < 0.05). Since EAI reflects the ability of proteins to adsorb at the oil-water interface and facilitate emulsion formation, these findings demonstrate that the extraction method substantially influenced the emulsifying activity of the recovered proteins. A similar trend was observed for ESI. Single-step BM produced the most stable emulsions (17.5 min), while two-step BM and single-step HPH exhibited lower ESI values of 11.1 and 12.6 min, respectively. No significant difference in ESI was observed between two-step BM and single-step HPH extracts (*p* > 0.05), although both were significantly lower than single-step BM (*p* < 0.05). The superior EAI and ESI values obtained for the single-step BM extract indicate an enhanced capacity to form and stabilize emulsions compared with the other extraction methods.

Collectively, these findings suggest that no single extraction method is universally optimal for all applications. The single-step BM approach produced protein extracts with superior emulsifying activity and stability, whereas the single-step HPH approach achieved the highest protein recovery yield. Consequently, the selection of an extraction strategy should be guided by the intended end use of the recovered protein. Applications prioritizing maximum protein recovery may favor the single-step HPH process, while applications requiring enhanced emulsification functionality may benefit from the single-step BM approach. Therefore, both protein recovery efficiency and functional performance should be considered when selecting an appropriate protein extraction method.

It should be noted that the recovered extracts contained approximately 66–68% protein, indicating the presence of substantial amounts of co-extracted non-protein components. Although the composition of these components was not determined, they may include carbohydrates, lipids, pigments, minerals, and other cellular constituents that could contribute to the observed water absorption, oil absorption, foaming, and emulsifying properties. Therefore, the functional characteristics reported in this study should be interpreted as properties of the protein-rich extracts as a whole rather than being attributed solely to the extracted proteins. Further compositional analyses are required to clarify the respective contributions of protein and non-protein components to the observed functionality.

While differences in protein solubilization, recovery, molecular weight distribution, and functional properties were experimentally observed, the underlying mechanisms responsible for these changes were not directly investigated. Cell disruption efficiency, protein hydrolysis, denaturation, and structural modifications were not quantified using complementary techniques. Consequently, mechanistic interpretations presented in this study should be regarded as hypotheses requiring further experimental verification.

## 4. Conclusions

This study demonstrated that integrating BM or HPH with alkaline treatment in a single-step process significantly enhanced microalgal protein extraction by simultaneously disrupting cells and solubilizing proteins. Among the evaluated methods, single-step HPH achieved the highest protein recovery (78.5%) at 0.1 M NaOH while maintaining protein purity (66.3%) comparable to that obtained using single-step BM and conventional two-step extraction comprising dry BM followed by alkaline treatment. In addition, both single-step approaches reduced alkali consumption relative to the two-step process. Although increasing NaOH concentration may further improve protein solubilization, SDS-PAGE analysis revealed changes in protein profiles above 0.1 M NaOH. The observed differences in functional properties demonstrate that extraction conditions influence not only protein recovery but also the application potential of the resulting protein ingredients. Therefore, process selection should be based on a balanced consideration of recovery efficiency and the retention of desirable functional attributes, as trade-offs may exist between maximizing protein recovery and functionality.

Overall, the findings demonstrate that the proposed single-step mechanochemical extraction process represents more than an incremental modification of the previously reported two-step dry BM-assisted extraction method. The key innovation lies in the integration of cell disruption and protein solubilization into a single processing step, resulting in higher protein recovery, shorter processing time and reduced process complexity. These attributes highlight the potential of the single-step mechanochemical approach as a promising process-intensification strategy for industrial microalgal protein production.

Future research should focus on elucidating the structural changes underlying the observed functional differences among extraction methods. Furthermore, the nutritional quality, including amino acid composition and in vitro protein digestibility, as well as sensory characteristics and performance, of these protein-rich extracts in food systems should be evaluated to support their industrial application as sustainable alternative protein ingredients.

## Figures and Tables

**Table 1:** The effect of the number of HPH passes on protein solubilization yield of *C. vulgaris*. Different letters indicate statistically significant differences at *p* < 0.05.

HPH Pass	Protein Solubilization Yield (%)
0	15.7 ± 0.48 ^a^
1	71.6 ± 2.07 ^b^
2	80.2 ± 4.73 ^c^
3	65.5 ± 0.90 ^b^
4	65.9 ± 2.57 ^b^
5	64.5 ± 4.47 ^b^

## Data Availability

The original contributions presented in this study are included in the article. Further inquiries can be directed to the corresponding author.
